# Inhibition of AGS Cancer Cell Proliferation following siRNA-Mediated Downregulation of *VEGFR2*

**DOI:** 10.22074/cellj.2016.4566

**Published:** 2016-08-24

**Authors:** Ali Zarei Mahmudabadi, Masoomeh Masoomi Karimi, Majid Bahabadi, Zahra Bagheri Hoseinabadi, Moslem JafariSani, Reza Ahmadi

**Affiliations:** 1 Department of Biochemical, Chemical Injuries Research Center, Baqiyatallah University of Medical Science, Tehran, Iran; 2 Department of Immunology, Faculty of Medicine, Torbat Heydariyeh University of Medical Sciences, Torbat Heydariyeh, Iran; 3 Department of Biochemistry, Qazvin University of Medical Sciences, Qazvin, Iran; 4 Department of Biochemistry, Rafsanjan University of Medical Sciences, Rafsanjan, Iran; 5 Department of Basic Sciences, School of Medicine, Shahroud University of Medical Sciences, Shahroud, Iran; 6 Biochemistry Center, Shahrekord University of Medical Sciences, Shahrekord, Iran

**Keywords:** VEGFR2, Downregulation, siRNA, Apoptosis

## Abstract

**Objective:**

Vascular endothelial growth factor (VEGF) and VEGF receptors (VEGFRs) play important roles in angiogenesis of different developmental mechanisms such as wound healing, embryogenesis and diseases, including different types of cancer. VEGFR2 is associated with cell proliferation, migration, and vascular permeability of endothelial cells. Blocking VEGF and its receptors is suggested as a therapeutic approach to prevent tumor growth. In this study, we aim to block VEGF signaling via small interfering RNA (siRNA) inhibition of VEGFR2.

**Materials and Methods:**

In this experimental study, we used the RNA interference (RNAi) mechanism to suppress expression of the *VEGFR2* gene. We conducted the 3-(4,5-di- methylthiazol-2-yl)-2,5-diphenyl-tetrazolium bromide (MTT) assay, real-time polymerase chain reaction (PCR), Western blot, and flow cytometry analyses of VEGFR2 expression.

**Results:**

Real-time PCR and Western blot results showed that VEGFR2 expression significantly downregulated. This suppression was followed by inhibition of cell prolifera- tion, reduction of viability, and induction of apoptosis in the cancer cells.

**Conclusion:**

These findings suggest that VEGFR2 has a role in cell proliferation and tumor growth. Accordingly, it is suggested that VEGFR2 can be a therapeutic target for controlling tumor growth and proliferation.

## Introduction

Vascular endothelial growth factor (VEGF) and its receptors are central regulators of angiogenesis during wound healing, reproduction, organ development, and embryogenesis as well as different diseases such as cancer and inflammatory diseases (1,2). They are secreted from glycoproteins that act as vital survival factors for endothelial cells and mediate cell proliferation, vascular permeability, and migration (3,4). The biological function of VEGF is mainly mediated via interaction with its receptors that belong to the family of tyrosine kinases-VEGFR1 (FLT1), VEGFR2 (KDR) and VEGFR3 (5). Although both VEGFR1 and VEGFR2 are found in endothelial cells, VEGFR1 is expressed in macrophages, hematopoietic stem cells, and tumor cells such as lung, breast, and pancreatic cancers, as well as hematopoietic malignancies (6). VEGFR1 has been well-described as a key regulator of migration as well as VEGFR2 signaling. VEGFR1 seems to be associated with accelerated proliferation of transformed cells and plays an important role in malignant cell growth (7). It has been shown that VEGFR2 induces expression of growth factors in liver sinusoidal endothelial cells (8). *VEGFR2* expression in certain cancers such as breast cancer showed a significant relation to high risk for metastasis and relapse. Hence, it is considered a marker for breast tumor aggressiveness (9). 

Recent studies show that VEGF and its receptors are crucial molecules in induction or inhibition of angiogenesis and growth of tumor cells (10). High level expression of *VEGF* stimulates angiogenesis while applying monoclonal antibodies inhibit or degrade VEGF and suppress the angiogenesis process (11). These findings highlight the issue that the VEGF pathway may be an important therapeutic target. Results from clinical trials using an aptamer (12) or an antibody fragment (13) that bind VEGF have already supported this concept. Another strategy to antagonize VEGF is to block the VEGFRs. A remarkable advantage of this approach is simultaneously blocking multiple VEGF family members (14). 

RNA interference (RNAi), a fundamental biological process by which cells regulate gene expression, acts through complementary basepairing with target mRNA and retrieves cellular RNases which in turn degrade mRNA transcripts (15). The RNAi strategy has developed rapidly from a basic scientific discovery to a powerful research tool and more recently, to a promising therapeutic approach (16). RNAi is now routinely used to evaluate gene function both *in vitro* and *in vivo*. Many innovative studies have reported the use of RNAi to investigate potential drug targets (17). Specific gene knockdown that can be achieved using RNAi as a therapeutic approach have made such therapies very attractive to many scientists as cure for different diseases. Small interfering RNAs (siRNAs) provide a useful means to selectively reduce the amount of mRNA transcripts and probe the function of gene products (18). In our previous study we have used siRNA to reduce *VEGFR1* expression *in vitro* (19,22). In the present study, we aimed to specifically downregulate *VEGFR2* expression in the ATCC® CRL1739™ human gastric carcinoma cells (AGS) using synthetic siRNA. 

## Materials and Methods

In this experimental study, we used the RNAi mechanism to suppress expression of the VEGFR2 gene. This project conducted according to approv- al from the Baqiyatallah University of Medical Sciences Ethical Committee.

### Materials

RPMI-1640, fetal bovine serum (FBS), penicillin-streptomycin (Pen/Strep), and trypsin enzyme were purchased from Gibco (USA). RNA extraction, cDNA synthesis and polymerase chain reaction (PCR) purification kits were obtained from Roche, Germany. Restriction enzymes were purchased from Jena Bioscience. 

### Methods

#### Small interfering RNA design

Anti-*VEGFR2* siRNAs were purchased from Takapuzist Gene Molecular Biotechnology Co., Ltd. (Iran). The siRNA was designed using an AsiDesigner (Bioinformatics Research Center, KRIBB) to target *VEGFR2* at the 5´-TAGCTGGGAATAGTAAAGC-3´ sequence. Sense and antisense sequenc- es were as follows for siRNA1: Si-sense1: 5´-GCUUUACUAUUCCCAGCUA-3´; and Si-antisense1: 5´-UAGCUGGGAAUAGUAAAGC-3´. Each siRNA was re-suspended in double distilled water and the stock solutions (20 μmol/L) were stored at 4˚C.

#### Cell culture

AGS cells (Pasteur Institute, Iran) were grown in RPMI-1640 medium that contained 10% FBS. Cells were incubated in a humidified 5% CO_2_ incubator at 37˚C for 48 hours. Viability of cells were examined by trypan blue, after which they were incubated under hypoxic conditions (3% O_2_, 5% CO_2_, 92% N_2_) for 24 hours to upregulate expression of the target gene.

### Transfection of small interfering RNA

AGS cells were cultured in RPMI-1640 medium. Briefly, 4×10^5^ cells were seeded onto six-well plates that contained antibiotic-free Dulbecco’s Modified Eagle Medium (DMEM, Gibco, USA). Plates were incubated overnight at 37˚C. For each well, 2 μl of siRNA (0.1 nmol/L) was mixed with 50 μl of DMEM. This mixture was then combined with a solution of 1 μl lipofectamine® 2000 (Invitrogen, USA) in 50 μl DMEM and incubated for 20 minutes at room temperature. Finally, a mixture that had a final concentration of 20 pmol/L for each siRNA was applied to the cells. After incubation for 4 hours at 37˚C, we replaced the media with fresh RPMI-1640 medium supplemented with serum and Pen/Strep (100 µg/ml). All tests were performed in triplicate at 24, 48, and 72 hours after siRNA transfection.

### Cell viability assay

The cells were seeded onto 96-well plates at a density of approximate 2×10^4^ cells per well and incubated at 37˚C in a 5% CO_2_ humid incubator for 24 hours. The 3-(4,5-dimethylthiazol-2-yl)-2,5- diphenyl-tetrazolium bromide (MTT) assay (23) was used to determine cell viability at 24, 48, and 72 hours after the cells were transfected with anti- VEGFR2 siRNA. We measured absorbance at 570 nm using a Quant Universal Microplate Spectrophotometer (BioTek, Winooski, VT).

### Colony formation assay

In the colony formation assay, we seeded the cells onto a 12-well plate at a density of 300 single cells per well after transfection. The medium was changed every three days. After approximately 10 days, most clones contained more than 50 cells. The clones were subsequently washed with 1X phosphate-buffered saline (PBS) and stained with crystal violet for approximately 5 minutes. Finally, the clones were imaged and quantified. The colony formation rate was calculated as the (number of clones)/(number of seeded cells)×100. 

### RNA extraction and cDNA synthesis

Total RNA was extracted from the cells using the RNX^TM^ plus solut Cinnagen,Iran )according to the manufacturer’s instructions. Briefly, 2 µg of total RNA was reverse transcribed and cDNA synthesized using a cDNA synthesis kit (Roche Co., Germany), according to the manufacturer’s instructions. The reverse transcription reaction for first strand cDNA synthesis was performed with 3-5 µg of purified total RNA with RevertAid™ Reverse Transcriptase (Fermentas, Canada) using oligo (dT)18 in a 20 µl total reaction mixture, according to the manufacturer’s instructions. 

### Real-time polymerase chain eaction analysis of VEGFR2 mRNA levels

mRNA expression levels of genes were estimated with the appropriate primers. The relative expression of each gene was assessed and compared to the housekeeping gene glyceraldehyde-3-phosphate dehydrogenase (* GAPDH*) with specific primers. All primers were designed using Primer Express® software (Applied Biosystems, USA). Amplifications were carried out using the following primers for

VEGFR2: F: 5´-CACTGGTTGTACCTCAGCAC-3´
  R: 5´-CGTACCAGAAGACACTTCGT-3´
GAPDH: F: 5´-GTGAACCATGAGAAGTATGACAA-3´
  R: 5´-CATGAGTCCTTCCACGATAC-3´.

Quantitative RT-PCR was performed using the 7500ABI system (Applied Biosystems, USA) in final reaction volumes of 20 µl with 20 ng cDNA, 10 µl of SYBR Green I master mix (Takara, Japan) and 200 nM of forward and reverse primers, according to the manufacturer’s instructions. The PCR reaction was performed as follows: initial denaturation of templates at 95˚C for 5 minutes, followed by 35 cycles of denaturation at 95˚C for 15 seconds, and annealing/extension at 60˚C for 30 seconds. Specificity of PCR products was examined by running on a 2% agarose gel to verify their size and dissociation curve analysis. We used serially diluted cDNA to obtain a standard curve and amplification efficiency for each primer of the gene transcript. For all gene expression analyses, the appropriate negative controls that contained no template controls were subjected to the same procedure in order to exclude or detect any possible contamination. All tests were repeated three times. 

### Western blot analysis

Western blot analysis was carried out for VEGFR2 using an anti-VEGFR2 antibody (Abcam Co.) as the primary antibody. The cells were collected and lysed by RIPA lysis buffer (Sigma-Aldrich Corp., St. Louis, MO, USA). Total protein was extracted and stored at -80˚C. The extracts were then mixed with 6× sodium dodecyl sulfate (SDS) buffer and boiled for 4 minutes. Samples were separated by 10% SDS-polyacrylamide gel electrophoresis and transferred to a polyvinylidene fluoride (PVDF) membrane. Membranes were blocked with 5% (w/v) skim milk in PBS that contained 0.1% Tween-20 for 1 hour at room temperature, washed with PBS, and probed with primary antibodies overnight at 4˚C. Membranes were washed again with PBS and incubated at room temperature with horseradish peroxidase (HRP)-conjugated anti-rabbit secondary antibodies (Abcam Co., UK) for 1 hour. Proteins were visualized with an enhanced chemiluminescence detection kit (Amersham Bioscience, Buckinghamshire, England). Actin (a goat polyclonal antibody) was used as the internal control. 

### Apoptosis assay

The number of apoptotic cells was calculated with an Annexin-V-PI detection kit (Abcam Co., UK). AGS cells at a density of 2×10^5^ were cultured, suspended in RPMI1640 with 10% FBS, and seeded in a 24-well flat-bottomed plate, then incubated for 24 hours at 37˚C. Cells were then transfected by anti-VEGFR2 siRNA. After incubation for 48 hours, the cells were collected and washed with PBS, after which PI and annexin V were added directly to the cell suspension in the binding buffer that consisted of 10 mM HEPES, 140 mM NaCl, and 2.5 mM CaCl_2_ at pH=7.4. The cells were incubated in the dark for 15 minutes at 37˚C, followed by flow cytometry analysis.

### Statistical analysis

The results were analyzed with one-way ANOVA followed by the t test using the Graphpad Prism 5.0 program and SPSS (SPSS, Chicago, IL, USA). A P≤0.05 was considered significant. Data were shown as mean ± SD. 

## Results

### Cell viability assay

**Fig.1:**
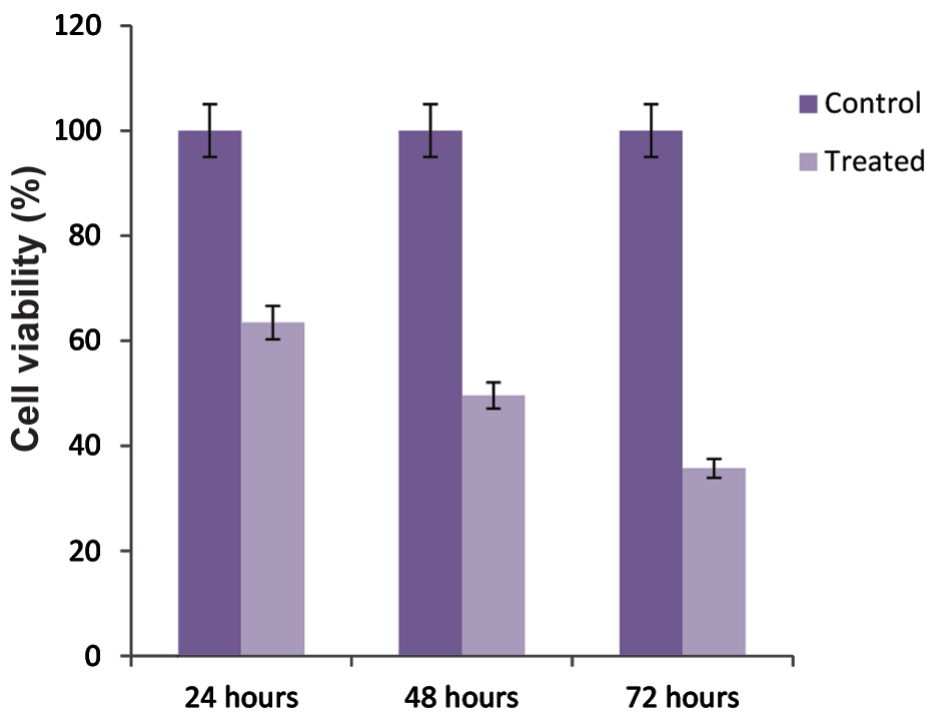
Survival ratios of AGS cells treated with anti-vascular endothelial growth factor 2 (anti-VEGFR2) small interfering RNA (siRNA). The 80% confluent cell cultures were treated with siRNA. Cell viability significantly reduced in a time-dependent manner. At 48 hours after treatment more than 50% of cells died. Results are the means of three independent experiments by the MTT assay (P≤0.05).

We conducted the MTT assay to evaluate the effect of anti-*VEGFR2* siRNA on viability of AGS cancer cells. AGS cells were transfected with anti-*VEGFR2* siRNA, then incubated for 24, 48, and 72 hours. The MTT assay results showed that using specific siRNA against *VEGFR2* reduced the viability of AGS cells compared with the control group in a time-dependent manner (Fig.1). The results showed the cytotoxicity of *VEGFR2* suppression to AGS cancer cells. Untreated cells were used as the control. 

### Colony formation assay

**Fig.2:**
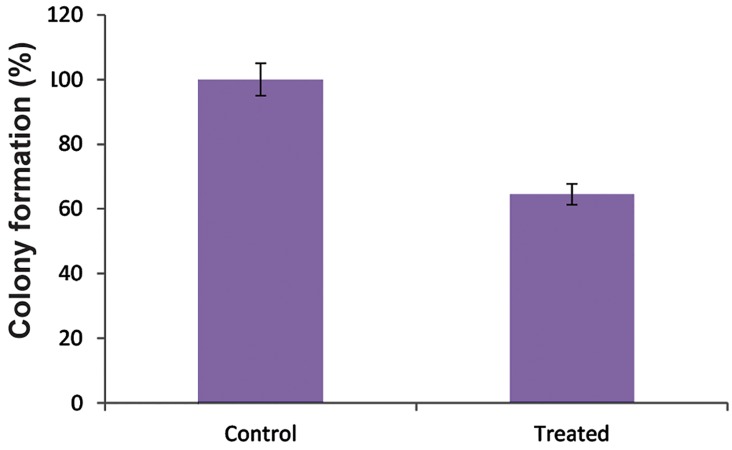
Colony formation ratio of AGS cells after transfection with anti-vascular endothelial growth factor 2 (anti-VEGFR2) small interfering RNA (siRNA) after 72 hours. The data showed significant reduction in colony formation. All experiments were performed in triplicate. Values are mean ± SD.

In order to further elucidate the effect of anti- *VEGFR2* siRNA AGS cell growth, we performed the colony formation assay at 24, 48, and 72 hours. Figure 2 shows that the colony formation rate of AGS cells transfected with anti-VEGFR2 siRNA reduced to approximately 65% compared to the control cells. These results showed that anti-VEGFR2 siRNA impaired proliferation of the AGS cell line. All experiments were performed in triplicate. 

### Real-time polymerase chain reaction for VEGFR2 mRNA 

**Fig.3:**
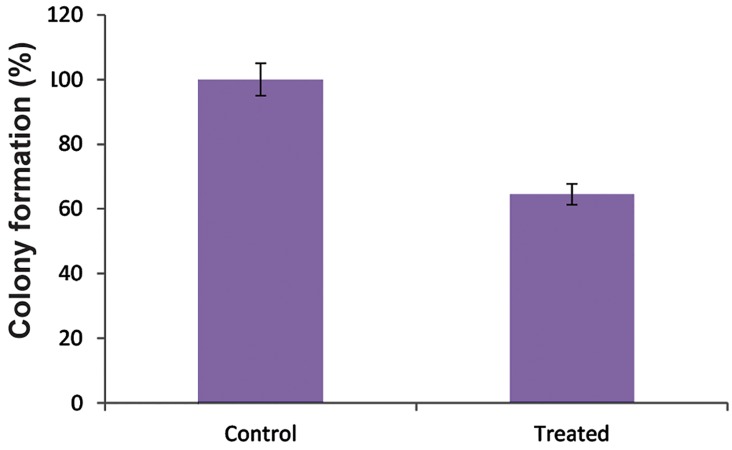
Quantitative analysis of vascular endothelial growth factor 2 (*VEGFR2*) gene expression levels downregulated in AGS cells 72 hours after treatment with anti-*VEGFR2* small interfering RNA (*siRNA*). Each real-time polymerase chain reaction (PCR) analysis was carried out at least in triplicate. Data are the fold change in relative expression compared with glyceraldehyde-3-phosphate dehydrogenase (*GAPDH*) based on the comparative Ct (2^-ΔΔCt^) method. Values are shown as mean ± SD.

The effect of anti-*VEGFR2* siRNA transfection on *VEGFR2* gene expression was analyzed by quantitative real-time PCR after 24, 48, and 72 hours. Total RNA was extracted from both transfected and control cells after treatment with siRNAs against *VEGFR2*. *GAPDH* was used as a reference to compare gene expression in different cells. Real-time PCR analysis revealed that the expression level of *VEGFR2* decreased significantly in cells treated with specific siRNAs compared to control cells without siRNA treatment (Fig.3). The results indicated that anti-*VEGFR2* application successfully decreased the *VEGFR2* mRNA level. 

### Western blot analysis

**Fig.4:**
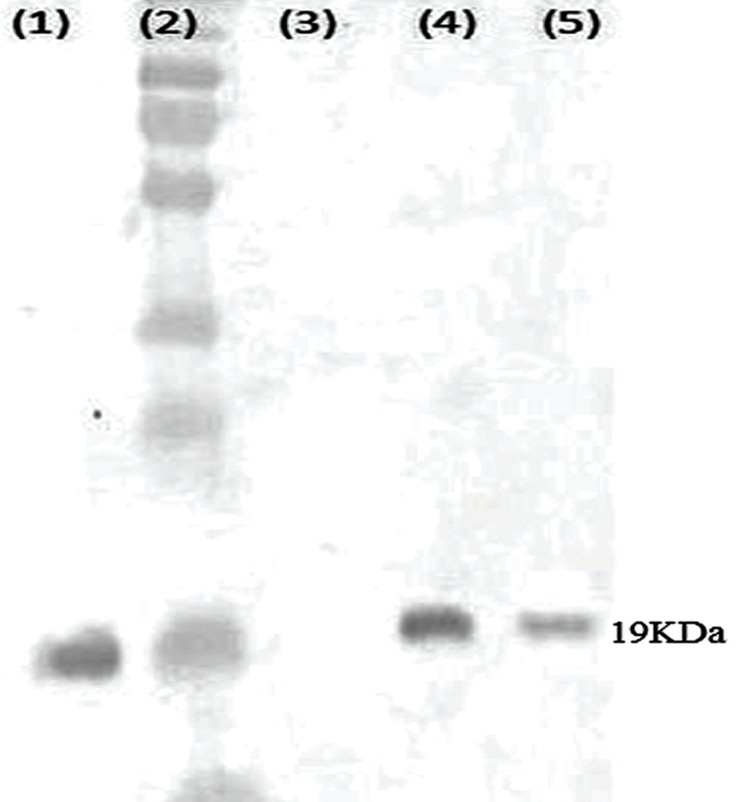
Analysis of the small interfering RNA (siRNA) effect on vascular endothelial growth factor 2 (VEGFR2) protein expressions in AGS cells according to Western blot after 72 hours. Lane 1; β-actin as the positive control, Lane 2; Protein marker, Lane 3; Negative control without protein loading, Lane 4; AGS cells without siRNA treatment, and Lane 5; AGS cells transfected with siRNA. As shown, a 19 KDa protein expressed in transfected cells without siRNA treatment. Transfection with siRNA significantly reduced VEGFR2 protein levels.

Total protein was extracted from cells and analyzed on sodium dodecyl sulfate-page (SDS-PAGE), followed by Western blot analysis to measure VEGFR2 protein levels in AGS cells after 24, 48, and 72 hours. As Figure 4 shows, un-transfected cells expressed a 19 kDa VEGFR2 band while cells transfected with siRNAs against VEGFR2 showed significant reduction of VEGFR2 protein level compared to the control group. This indicated that use of anti-VEGFR2 siRNA specifically targeted VEGFR2 mRNA and influenced its protein production. We used β-actin as the positive control in this experiment. 

### Apoptosis assay

**Fig.5:**
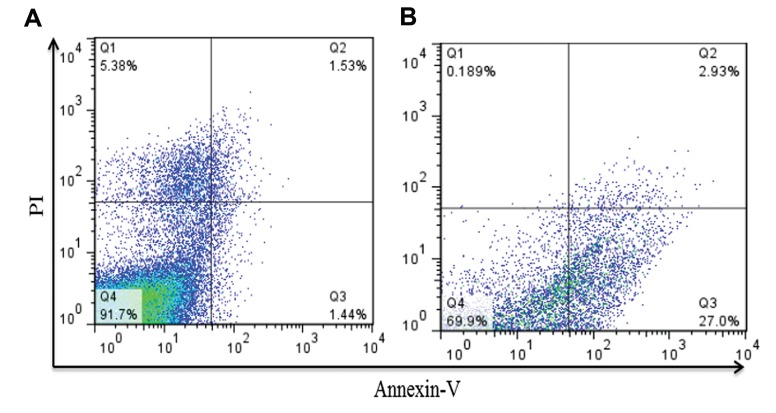
Flow cytometry analysis of AGS cells stained with Annexin V-FITC and PI. A. Untreated cells were used as controls and B. Cells underwent apoptosis induced by anti-vascular endothelial growth factor 2 (anti-VEGFR2) small interfering RNA (siRNA) after 72 hours. Diagrams Q1 to Q4 represent necrotic, early apoptotic, late apoptotic and live cells, respectively. Treated cells significantly increased in the rate of early and late apoptosis compared with the control.

We used an Annexin-V-PI kit to measure the number of apoptotic cells after transfection with siRNA against *VEGFR2* after 24, 48, and 72 hours. Cells seeded in a 24-well plate were transfected with anti-*VEGFR2* siRNA. Cells were subsequently treated with Annexin V-FITC and PI, then analyzed by flow cytometer. Figure 5 shows that the number of apoptotic cells significantly reduced following transfection with siRNA compared with the control group. Total apoptosis increased from approximately 2.93% (Q2 as early apoptotic) to 27.0% (Q3 as late apoptotic) in the treated group compared to the control group, which supported the cell viability assay results. 

## Discussion

The key roles of VEGF and its receptor VEGFR1 and VEGFR2 in tumor angiogenesis and tumor growth are well-established. Activation of VEGFR2 leads to the activation of downstream signaling pathways (24). In contrast, the function of VEGFR1 is still poorly determined (25). VEGFR1 has been shown to be associated with tumor growth, tumor cell activation, and metastasis (26). Studies reported VEGFR1 expression in many tumors including breast, lung, and gastric cancers (27,29). Upregulation of serum VEGFR1 has been shown in gastric cancer patients (30). Blocking VEGF is an important way to control angiogenesis and cancer growth. It was previously demonstrated that VEGF, VEGFR1 and VEGFR2 co-expressed in gastric adenocarcinoma MGC803 cells as well as eight gastric cancer cell lines that included AGS-1, RF-1 and RF-48, as well as gastric tumor specimens (31,35). 

There are different anti-VEGF agents used to control angiogenesis include chemicals and antibodies (35,36). Inhibition of its receptors is another strategy to antagonize VEGF which in turn can simultaneously block several VEGF family members. VEGFRs have been considered for cancer therapy and production of anti-cancer drugs (37). Several VEGFR inhibitors have been developed such as SU6668, ZD6474, PTK787 compounds and mono-clonal antibodies (38). Hwang et al. (30) reported that blockage of VEGFR1 and VEGFR2 with concomitant paclitaxel increased cell cytotoxicity of TUBB3-expressing gastric cancer cells. They demonstrated that AGS cell cytotoxicity was more obvious when cells were treated simultaneously with paclitaxel, anti-VEGFR1, and anti-VEGFR2. From these results, we suggested that the AGS cells expressed VEGFR1. In the present study, we suppressed the expression of *VEGFR2* in the AGS cell line using anti-VEGFR2 siRNA. Real-time PCR analysis of mRNA levels showed that siRNA efficiently decreased *VEGFR2* expression compared to control cells. Western blot analysis showed that after the use of siRNA against VEGFR2, its protein level significantly reduced compared to the control group. The colony formation assay and MTT assay results showed that downregulation of VEGFR2 inhibited cancer cell growth and significantly reduced cell viability. Also, apoptosis assay results indicated significant induction of cell death. However, in addition to inhibition of cell growth, our results suggested that suppression of VEGFR2 could significantly drive cells to apoptosis. Some investigators have reported that VEGFR2 suppression is not adequate to hinder tumor growth without the combined inhibition of VEGFR2 (39,40). Of note, VEGFR2 signaling within tumor cells was previously shown to regulate growth and survival of several mouse tumor models and cell lines (31,41). Some reports suggested a possible role for VEGFR2 in survival and growth of cancer cell lines such as pancreatic and colorectal cancer cell lines (42). Inhibition of VEGFR2 signaling using hyper methylation showed tumor growth inhibition and decreased survival of cancer cells in some tumor models (35). 

Overall, the current research indicated that blocking VEGFR2 using RNAi suppressed cell proliferation in AGS cells, as well as induction of apoptosis in AGS cells. 

## Conclusion

Blocking VEGF and its receptors have been proposed as a therapeutic approach for inhibition of cancer growth. In the present study, we used RNAi to downregulate expression of the *VEGFR2* gene using specific siRNAs in AGS cells. The results showed that suppression of VEGFR2 inhibited AGS cell proliferation and drove them to apoptosis. 
